# Prevalence and Associated Factors of Elder Mistreatment in a Rural Community in People's Republic of China: A Cross-Sectional Study

**DOI:** 10.1371/journal.pone.0033857

**Published:** 2012-03-20

**Authors:** Li Wu, Hui Chen, Yang Hu, Huiyun Xiang, Xiang Yu, Tao Zhang, Zhongqiang Cao, Youjie Wang

**Affiliations:** 1 Department of Maternal and Child Health, School of Public Health, Tongji Medical College, Huazhong University of Science and Technology, Wuhan, China; 2 Tongji Center of Injury Prevention, Tongji Medical College, Huazhong University of Science and Technology, Wuhan, China; 3 Center for Injury Research and Policy, The Ohio State University, Columbus, Ohio, United States of America; Cardiff University, United Kingdom

## Abstract

**Background:**

Current knowledge about elder mistreatment is mainly derived from studies done in Western countries, which indicate that this problem is related to risk factors such as a shared living situation, social isolation, disease burden, and caregiver strain. We know little about prevalence and risk factors for elder mistreatment and mistreatment subtypes in rural China where the elder population is the most vulnerable.

**Methods:**

In 2010, we conducted a cross-sectional survey among older adults aged 60 or older in three rural communities in Macheng, a city in Hubei province, China. Of 2245 people initially identified, 2039 were available for interview and this was completed in 2000. A structured questionnaire was used to collect data regarding mistreatment and covariates. Logistic regression analysis was used to identify factors related to elder mistreatment and subtypes of mistreatment.

**Results:**

Elder mistreatment was reported by 36.2% (95% CI: 34.1%–38.3%) of the participants. Prevalence rates of psychological mistreatment, caregiver neglect, physical mistreatment, and financial mistreatment were 27.3% (95% CI: 25.3%–29.2%), 15.8% (95% CI: 14.2%–17.4%), 4.9% (95% CI: 3.9%–5.8%) and 2.0% (95% CI: 1.3%–2.6%), respectively. The multivariate logistic regression analysis revealed that depression, being widowed/divorced/single/separated, having a physical disability, having a labor intensive job, depending solely on self-made income, and living alone were risk factors for elder mistreatment. Different types of elder mistreatment were associated with different risk factors, and depression was the consistent risk factor for the three most common mistreatment subtypes.

**Conclusion:**

Older adults in rural China self-report a higher rate of mistreatment than their counterparts in Western countries. Depression is a main risk factor associated with most subtypes of mistreatment. Our findings suggest that prevention and management of elder mistreatment is a challenge facing a rapidly aging Chinese population.

## Introduction

Elder mistreatment is an important public health issue, and prior studies have suggested that such mistreatment can cause significant adverse health outcomes [1], [2]. At present there is no generally valid definition of elder mistreatment around the world. The National Research Council (NRC) report “Elder Mistreatment: Abuse, Neglect and Exploitation in an Aging America” defined elder mistreatment as “intentional actions that cause harm or create a serious risk of harm, whether or not intended, to a vulnerable elder by a caregiver or other person who stands in a trust relationship to the elder or failure by a caregiver to satisfy the elder's basic needs or to protect the elder from harm” [3].

Epidemiological studies have revealed that mistreatment of the older people is common in Western countries. Several studies from developed countries suggest that the prevalence rate is between 2% and 10% [4]. In Western countries, mistreatment of older persons is related to a shared living situation, social isolation, disease burden, and caregiver strain [5]. However, few studies have been done in developing countries on elder mistreatment. Little is known about the prevalence of and risk factors for elder mistreatment in China. People believe that elder mistreatment is not common in China because of strong family ties in that culture. The traditional Chinese value of filial piety requires adult children to love, respect, and care for their parents. However, a clinic-based population study has suggested that elder mistreatment in China is alarmingly common in the urban elderly population [6]. Dong et al. study showed that elder mistreatment is common and considered unacceptable in Chinese culture [7]. Studies have also revealed that elder mistreatment is a serious issue in Hong Kong society [8]. Since the economic reforms of 1978, China has experienced tremendous socioeconomic changes. Its rapid growth has been accompanied by substantial changes in traditional Chinese values. Old customs like the obligation to venerate and care for the older people are breaking down. Most Chinese elderly people rely on their children for care and financial support, especially those in rural areas who have no adequate pension or medical care that is largely available for the urban older people. The lack of financial support and dependence on children make the rural older people in China a vulnerable population for elder mistreatment. Furthermore, migration of young people to cities in search of jobs has greatly weakened the family's perceived obligation of caring for their elder members. As a result, the vulnerability of older persons has increased. However, no research about elder mistreatment in rural areas of China has been reported. To fill this knowledge gap, we used the definition of elder mistreatment of the National Research Council Panel to Review Risk and Prevalence of Elder Abuse and Neglect to measure elder mistreatment. We examined the prevalence of elder mistreatment as well as possible risk factors and mistreatment subtypes in a rural community of China. We restricted the definition of elder mistreatment here to psychological, financial, or physical mistreatment as well as neglect by a family member, in-laws, or relatives.

## Methods

### Study design and participants

This study was conducted in Macheng, a city located in northeastern Hubei province, China. According to official government data, this city has a population of 1,200,000, and 70% live in rural areas. Residents aged 60 or older accounted for 14.2% of the population in 2010.

We used a two-stage cluster sampling to select the study subjects. In the first stage, we selected 3 rural districts (Longchi, Nanhu and Gulou) randomly from 19 districts of Macheng. In the second stage, we selected 17 rural villages randomly from a total of 34 villages in the 3 districts. The inclusion criterion was all adults aged 60 years or older living in the selected 17 villages. We identified 2,245 qualified adults from the official residency registration lists of the villages. The exclusion criteria were being cognitively impaired, deaf, or unavailable for the interview. Cognitive impairment was not formally assessed but was based on the interviewer's judgment of the person's ability to provide consistent answers. There were 206 older adults who were unavailable for the interview because they were staying somewhere else. Finally, a total of 2,039 were interviewed. Among them 2,000 were successfully interviewed, while 28 did not complete the interview because of impaired cognition, confusion, or inconsistent answers to questions. There were 5 older adults who were deaf, and 6 eligible adults refused to be interviewed. To maximize response rate, each participant received 15 Yuan RMB (about 2.5 US Dollars) (equivalent to about one quarter of the monthly pension).

### Data collection and study variables

Data collection for our study was performed between November 1 and November 30, 2010. The questionnaire was pretested with 10 older adults living in the investigated villages to validate the clarity of meaning and appropriate use of the language. The interviewer team comprised 3 researchers, 4 PhD students and 5 Master's Degree students from our School of Public Health. All interviewers were medically qualified, and they received one-day training on the interview protocol and skills needed to ask personal questions. The face-to-face interview was conducted by the trained interviewer at the home of the older people. Trained interviewers approached the eligible participants, explained in detail the purpose of this survey, and asked whether they would like to participate. Because most of the elderly people were illiterate, oral informed consent for the interview was obtained from each participant. To protect participants' privacy and encourage them to report mistreatment, family members were asked not to be present in the interviewing room. This study protocol was approved by the ethics committee of Tongji Medical College, Huazhong University of Science and Technology.

We collected participant information including age, gender, education, marital status, number of children, source of income, living arrangement, physical disability, chronic diseases, and labor intensity. Information about chronic diseases was obtained by asking whether they had a history of physician-diagnosed diseases including hypertension, diabetes, cardiovascular disease, stroke, chronic bronchitis, emphysema, asthma, COPD, chronic liver, lung, or stomach disease, or malignant tumor. Physical disability was assessed by the interviewer based on whether the participant had impairments including upper and lower limb, spinal, or vision disabilities due to disease, trauma, or birth defect. People with a hearing disability were excluded from the interview. Age was grouped into three categories: 60–69 (reference category), 70–79, and 80 or older. Education was categorized as 5 years or less (reference category), and 6 years or more of schooling. Marital status was categorized as married (reference category) and widowed/ divorced/ single/ separated. Number of children was categorized as 0 (reference category), 1–4, and number of children 5 or more. Source of income was categorized as depending solely on self-made income (reference category), depending partially on self-made income, and depending solely on children. Living arrangement was categorized as living alone (reference category), living with spouse, living with spouse and children, and living with other family members.

### Elder mistreatment assessment

We selected and modified items from two well validated instruments for elder abuse: the Hwalek–Sengstock Elder Abuse Screening Test [9] and the Vulnerability to Abuse Screening Scale [10]. The study further considered the issues of elder mistreatment in Chinese culture. In order to be more specific in further exploring elder mistreatment in this cultural context, we modified the items in the instrument. We defined physical mistreatment as a willful infliction of hitting, kicking, pushing, slapping, burning or other show of force resulting in physical harm and pain. For example, we asked “Is there anyone in your family who hits, kicks, pushes, or slaps you?” We defined psychological or emotional mistreatment as acts done with the intention of causing emotional pain or injury. For example, we asked “Has anyone close to you called you names or put you down or made you feel bad recently?” We defined caregiver neglect as the failure to meet an elder's basic needs. For example, the question for caregiver neglect included questions such as “Is there anyone to take care of you when you are sick?” We defined financial mistreatment as illegally misusing an elder's money, property or assets. For example, the question for caregiver neglect included questions such as “Has anyone taken things that belong to you without your permission?” A yes to any of the screening questions within the previous 12 months was considered to be self-reported elder mistreatment. The elder mistreatment scale has an internal consistency α of 0.75 in this study, and factorial analysis resulted in these four factors, which explained 52.9% of the total variance.

### Depression evaluation

Depression was assessed based on the fifteen-question Geriatric Depression Scale (GDS-15) [11]. Respondents are asked to indicate whether they have experienced the symptoms described during the past week using the yes/no format. This study used the Chinese version in which a score greater than 8 suggests depression [12]. The GDS-15 was evaluated in Chinese people aged 60 or older and found reliable, with satisfactory internal consistency (Cronbach α = 0.80) and test-retest reliability (r = 0.73) [13].

### Statistical analysis

Data were analyzed using the statistics package SPSS 11.0. A descriptive analysis was performed on all study variables, using Mean ± SD for quantitative variables and absolute and relative frequencies for qualitative variables. Differences between means of two groups were tested using the Student *t-*test. The χ^2^ test was used to measure associations of each study variable with elder mistreatment. Each variable that was significantly associated based on results of the χ^2^ test was included in a logistic regression model to examine the independent effect for reported elder mistreatment. Odd ratios (ORs) and 95% confidence intervals (CIs) for each variable were obtained from the logistic regression model. Wald χ^2^ statistics and *P* values were used to evaluate the significance of individual model parameters, and the Hosmer-Lemeshow goodness-of-fit χ^2^ test was employed to assess the overall fit of logistic models. In this study, differences with a *P* value less than 0.05 were considered statistically significant.

## Results

### Participant characteristics

Of the available 2,039 older inhabitants, we excluded 39 from the analysis because of incomplete questionnaires. Of the 2,000 elder adults analyzed for our study, 801 (40.1%) were men, and 1,199 (59.9%) were women. Their age ranged from 60 to 93 years, with a mean age of 68.8 years (SD = 6.6). In this study sample, 63.4% (n = 1,268) were married, and 36.6% (n = 632) were widowed/ divorced/ single/ separated. Of the total, 81.0% (n = 1,620) had 5 years or less of schooling, and 19.0% (n = 380) had 6 years or more of schooling. In terms of living arrangement, 17.0% (n = 339) lived alone, 37.2% (n = 744) lived with a spouse, 41.1% (n = 822) lived with a spouse and children, and 4.8% (n = 95) lived with other family members. Financially, 41.1% (n = 821) depended solely on self-made income, 42.1% (n = 841) depended partially on self-made income, and 16.9% (n = 338) depended on financial support from children. Chronic medical conditions were reported by 60.8% (n = 1,216), and physical disability was reported by 8.3% (n = 166) of participants Subjects' characteristics stratified according to sex was present in Table 1.

**Table 1:** Subjects' characteristics stratified according to sex.

Characteristics	Male	Female	*P*
**Age(years),Mean ± SD**	69.0±6.5	68.7±6.7	>0.05
**Age, n (%)**			
60∼	453(56.6)	708(59.0)	>0.05
70∼	287(35.8)	406(33.9)	
≥80	61(7.6)	85(7.1)	
**Number of children, Mean ± SD**	3.3±1.5	3.5±1.4	<0.01
**Number of children (%)**			
0	20(2.5)	11(0.9)	<0.05
1∼4	621(77.5)	937(78.1)	
≥5	160(20.0)	251(21.0)	
**Marital status, n (%)**			
married	570(71.2)	698(58.2)	<0.01
widowed/divorced/single/separated	231(28.8)	501(41.8)	
**Education, n (%)**			
≤5 years	495(61.8)	1125(93.8)	<0.01
≥6 years	306(38.2)	74(6.2)	
**Living arrangement, n (%)**			
living alone	126(15.7)	213(17.8)	<0.05
living with spouse	345(43.1)	399(33.3)	
living with spouse and children	294(36.7)	528(44.0)	
living with other family members	36(4.5)	59(4.9)	
**Living source, n (%)**			
depending solely on self-made income	381(47.6)	440(36.7)	<0.01
depending partially on self-made income	329(41.1)	512(42.7)	
depending solely on children	91(11.4)	247(20.6)	
**Chronic disease, n (%)**			
yes	481(60.1)	735(61.3)	>0.05
no	320(39.9)	464(38.7)	
**Physical disability, n (%)**			
yes	73(9.11)	93(7.8)	>0.05
no	728(90.89)	1106(90.2)	

SD: Standard Deviation.

### Prevalence of various types of elder mistreatment

**Figure 1:**
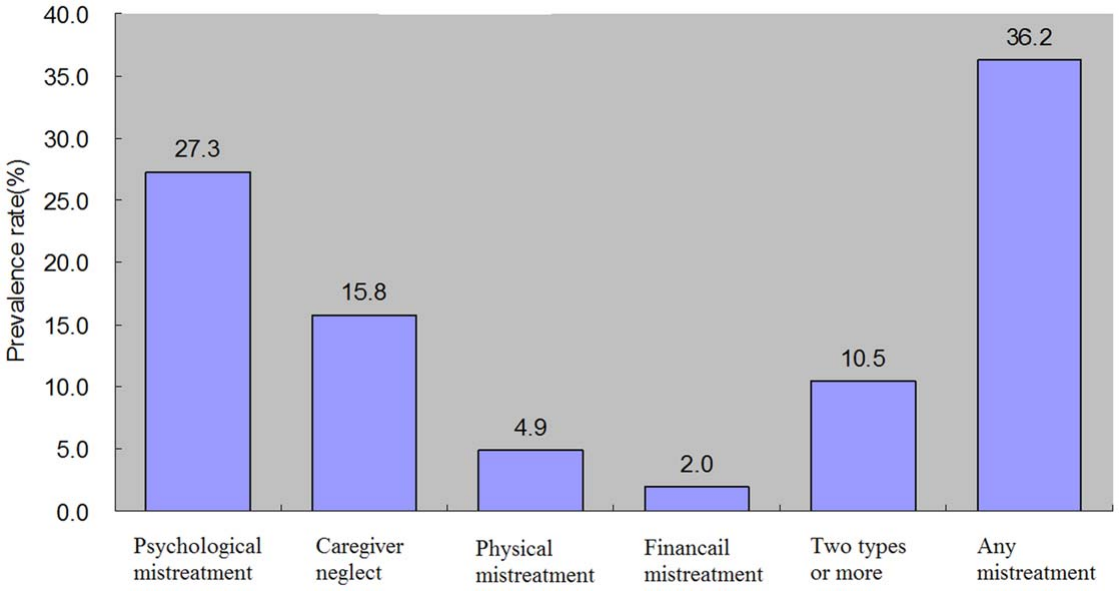
Prevalence rates of various types of elder mistreatment.

Among 2,000 participants, 724 (36.2%: 95% CI 34.1%–38.3%) reported that they had experienced at least one type of mistreatment (physical mistreatment, emotional mistreatment, caregiver neglect, or financial mistreatment) in the past year. Reported prevalence number of emotional mistreatment, caregiver neglect, physical mistreatment, and financial exploitation were 546 (27.3%: 95% CI 25.3%–29.2%), 316 (15.8%: 95% CI 14.2%–17.4%), 98 (4.9%: 95% CI 3.9%–5.8%) and 39 (2.0%: 95% CI 1.3%–2.6%) respectively. In our survey, 210 (10.5%: 95% CI 9.2%–11.8%) of elders reported two or more types of mistreatment (Figure 1).

### Characteristics of the elder mistreatment group

The characteristics of those who reported mistreatment were compared with the general population using the chi-square test (Table 2). Results demonstrated that elder mistreatment was significantly associated with being widowed/ divorced/ single/ separated, 5 years or less of school education, living alone, depending solely on self-made income, having chronic disease, physical disability, high labor intensity, and depression. Table 2 also shows frequencies of the different subtypes of elder mistreatment, grouped according to the different variables studied. Physical abuse was significantly associated with being solely dependent on self-made income and depression. Psychological abuse was significantly associated with being older, widowed/ divorced/ single/ separated, 5 years or less of schooling, living alone, being solely dependent on self-made income, having chronic disease, physical disability, high labor intensity, and depression. Caregiver neglect was significantly associated with being a male, widowed/ divorced/ single/ separated, living alone, depending solely on self-made income, high labor intensity, and depression. Financial mistreatment was associated with high labor intensity, physical disability, and depression. Depression was the factor consistently related to all subtypes of mistreatment.

**Table 2:** Prevalence of any mistreatment and subtypes of of elder mistreatment.

Characteristics	Any mistreatment n (%)	Physical n (%)	Psychological n (%)	Neglect n (%)	Financial n (%)
**Sex**					
male	293(36.6)	34(4.2)	206(25.7)	151(18.8)*	16(2.0)
female	431(36.0)	64(5.3)	340(28.4)	165(13.8)	23(1.9)
**Age**					
60∼	404(34.8)	59(5.1)	285(24.6)*	183(15.8)	25(2.2)
70∼	260(37.5)	29(4.2)	210(30.3)	105(15.2)	9(1.3)
≥80	60(41.1)	10(6.8)	51(34.9)	28(19.2)	5(3.4)
**Number of children**					
0	11(1.5)	0(0.0)	9(1.6)	5(1.6)	0(0.0)
1∼4	558(77.1)	80(81.6)	422(77.3)	243(76.9)	30(76.9)
≥5	155(21.4)	18(18.4)	115(21.1)	68(21.5)	9(23.1)
**Marital status**					
married	390(30.8)*	54(4.3)	265(20.9)*	183(14.4)#	24(1.9)
widowed/divorced/single/separated	334(45.6)	44(6.0)	281(38.4)	133(18.2)	15(2.1)
**Education**					
≤5 years	605(37.4)#	86(5.3)	463(28.6)*	257(15.9)	32(2.0)
≥6 years	119(31.3)	12(3.2)	83(21.8)	59(15.5)	7(1.8)
**Living arrangement**					
living alone	175(51.6)*	24(7.1)	153(45.1)*	73(21.5)*	9(2.6)
living with spouse	242(32.5)	32(4.3)	165(22.2)	105(14.1)	13(1.8)
living with spouse and children	275(33.4)	37(4.5)	212(25.8)	117(14.2)	16(1.9)
living with other family members	32(33.7)	5(5.3)	16(16.8)	21(22.1)	1(1.0)
**Living source**					
depending solely on self-made income	352(42.9)*	58(7.1)*	282(34.4)*	159(19.4)*	21(2.6)
depending partially on self-made income	260(30.9)	27(3.2)	174(20.7)	113(13.4)	14(1.7)
depending solely on children	112(33.1)	13(3.8)	90(26.6)	44(13.0)	4(1.2)
**Chronic disease**					
no	262(33.4)#	37(4.7)	189(24.1)#	130(16.6)	11(1.4)
yes	462(38.0)	61(5.0)	357(29.4)	186(15.3)	28(2.3)
**Physical disability**					
no	641(35.0)*	87(4.7)	477(26.0)*	286(15.6)	32(1.7)#
yes	83(50.0)	11(6.6)	69(41.6)	30(18.1)	7(4.2)
**Labor intensity**					
low	251(33.1)#	34(4.5)	203(26.7)#	91(12.0)*	7(0.9)*
moderate	254(35.7)	28(3.9)	175(24.6)	125(17.6)	14(2.0)
high	212(41.2)	34(4.5)	162(31.5)	98(19.0)	18(3.5)
**Depression**					
no	495(29.4)*	65(3.9)*	334(19.8)*	226(13.4)*	26(1.5)*
yes	229(72.5)	33(11.4)	212(67.1)	90(28.5)	13(4.1)

* *p*<0.01,

# *p*<0.05.

To control for confounding factors, a stepwise multiple logistic regression analysis was performed using elder mistreatment as the dependent variable, and all variables that reached a *P* value less than 0.05 in the chi-square test were examined as the independent variables. Results showed that elder mistreatment was significantly associated with being widowed/ divorced/ single/ separated, living alone, having a physical disability, depending on self-made income, labor intensity, and depression (Table 3).

**Table 3:** Stepwise multivariate logistic regression analysis of ORs for elder mistreatment among older people.

Variables	β	SE	Wald χ^2^	*P*	OR (95%CI)
**Marital status**					
married					1
widowed/divorced/single/separated	0.6	0.1	18.3	<0.01	1.8 (1.4∼2.4)
**Physical disability**					
no					1
yes	0.4	0.2	5.5	<0.05	1.5 (1.1∼2.2)
**Living arrangement**					
living alone					1
living with spouse and children	−0.4	0.2	5.4	<0.05	0.7 (0.5∼0.9)
**Living source**					
depending solely on self-made income					1
depending partially on self-made income	−0.3	0.1	7.2	<0.01	0.7 (0.6∼0.9)
depending solely on children	−0.5	0.2	8.5	<0.01	0.6 (0.4∼0.8)
**Labor intensity**					
low					1
moderate	0.3	0.1	4.7	<0.05	1.3 (1.0∼1.7)
high	0.4	0.1	7.1	<0.01	1.4 (1.1∼1.9)
**Depression**					
no					1
yes	1.7	0.1	140.7	<0.01	5.5 (4.1∼7.3)

Only statistically significant values are reported; OR: Odds Ratio, CI: Confidence Interval, β: parameter estimate, SE: standard error.

### Factors associated with elder mistreatment subtypes

We conducted multivariate logistic regression to identify the relative contribution of each factor to each subtype of elder mistreatment. Table 4 demonstrates that depression was associated with higher risk of self-reported physical mistreatment, while depression, physical disability, being widowed/ divorced /single/ separated, having chronic diseases, living alone, and depending on self-made income were associated with higher risk of psychological mistreatment. The variables associated with risk of caregiver neglect were depression, having a labor-intensive job, and being a male. The variables associated with risk of financial mistreatment were physical disability and having a labor-intensive job. (Table 4).

**Table 4:** The multivariate logistic regression analysis for each type of elder mistreatment.

Variables	OR(95% CI)			
	Physical	Psychological	Neglect	Financial
**Sex**				
male	–	–	1	–
female	–	–	0.6(0.5∼0.8)	–
**Marital status**				
married	–	1	–	–
widowed/divorced/single/separated	–	2.1(1.5∼2.8)	–	–
**Living arrangement**				
living alone	–	1	–	–
living with spouse	–	0.6(0.4∼0.9)	–	–
living with spouse and children	–	0.7(0.4∼0.9)	–	–
living with other family members	–	0.5(0.3∼0.9)	–	–
**Living source**				
depending solely on self-made income	–	1	–	–
depending partially on self-made income	–	0.6(0.5∼0.8)	–	–
depending solely on from children	–	0.5(0.3∼0.7)	–	–
**Chronic disease**				
no	–	1	–	–
yes	–	1.3(1.0∼1.6)	–	–
**Physical disability**				
no	–	1	–	1
yes	–	1.5(1.1∼2.2)	–	2.8(1.2∼6.6)
**Labor intensity**				
low	–	–	1	1
moderate	–	–	1.6(1.1∼2.3)	–
high	–	–	1.8(1.3∼2.4)	2.6(1.4∼5.0)
**Depression**				
no	1	1	1	–
yes	6.3(4.8∼8.3)	6.9(5.2∼9.1)	2.6(1.9∼3.5)	-

OR: Odds Ratio, CI: Confidence Interval.

## Discussion

Elder mistreatment is now recognized internationally as a serious public health problem. However, current scientific knowledge regarding this problem in China is still lacking. To our knowledge, this is the first population-based study examining the prevalence of elder mistreatment in a rural community in China. In this study, we found that elder mistreatment was common, with an estimated prevalence of 36.2% in the previous 12 months. The present estimate of overall elder mistreatment was much higher than estimates obtained from studies done in Western countries [14], [15]. In addition to the overall prevalence, results from this study confirm the findings of other studies that indicate psychological mistreatment and neglect are the most common types of elder mistreatment [5], [16], [17].

Elder mistreatment, like any other form of family violence, is extremely complex, and various factors contribute to its occurrence. In our study, we found that several factors were associated with elder mistreatment. Depression, being widowed/ divorced/ single/ separated, having physical disability, having a labor-intensive job, depending on self-made income, and living alone significantly increased the risk of elder mistreatment. Our findings are similar to a study done by Dong et al [18], in which depression, as defined by positive responses on five questions of the Geriatric Depression Scale, was significantly associated with elder mistreatment. In contrast to previous research, our study showed that elder people living alone were more likely to fall victim to mistreatment. Most studies have indicated that a shared living situation is a major risk factor for elder abuse and that people living alone are at lowest risk [19]–[21]. There are increased opportunities for contact and thus perhaps conflict and tension in a co-residential living arrangement. It is a Chinese cultural norm and value for adult children to take responsibility for providing care for their older parents, and elderly people prefer to live with adult children (particularly the eldest son and his wife and children) for emotional, physical, and financial support [22]. Living alone can make older people feel isolated and neglected by their families and relatives. Studies have shown that living alone is associated with lower subjective well-being among the Chinese older people [23]. On the other hand, living alone may be the result of elder mistreatment rather than a risk factor. Selfish adult children might be tired of taking care of their older parents and therefore force them to live alone. This phenomenon was frequently observed in our field survey.

Depending on self-made income was related to a higher level of mistreatment. A possible explanation for this result is that the abuser might have financial difficulties and extort the elder adult's property, resulting in mistreatment [24]. It has been reported that having adult children depending on elderly parents for housing and financial assistance increases the risk of elder mistreatment [25]. Unlike developed countries where social security systems are well established, China does not have a good welfare or social security system for its elders. Especially in rural areas where the majority of Chinese older people reside, family is usually the main or only source of financial support. Similar to living alone, financial independence might be the result of elder mistreatment. If adult children neglect elder parents' basic needs, those elders have to depend on self-made income, which may be viewed by the elder as a mistreatment. Our study also showed that high labor intensity was associated with higher risk of elder mistreatment. A labor intensive job held by elder adults may be associated with a dependence on self-made income or insufficient financial support from children.

It was reported that older women were at higher risk of elder mistreatment compared with their male counterparts [26], [27]. Rural older women in China have lower socio-economic status in family and tend to dependence on financial and emotional support from their children [28], thereby might impose a higher risk of mistreatment on them. But we did not find the gender difference of elder mistreatment in this study. We speculated the possible reason for this finding was that older women rather than older man in China were responsible for domestic chores, such as cooking, cleaning, taking care of grandchildren, which might win the respect from their children, and in turn, reduce the mistreatment risk.

In this study, we found that risk factors varied somewhat by mistreatment type, but the three most common types of elder mistreatment were associated with depression, even after we controlled for the effects of all other variables that were tested. This finding was consistent with previous studies [29], [30]. Older adults who reported depression had increased risk of suffering mistreatment.

Our study demonstrated that physical disability and chronic diseases were independent variables for psychological mistreatment. Caring for elder parents with physical disabilities and chronic diseases requires substantial support and often personal sacrifice on the part of the caregivers and other family members [31]. This can put undue stress on the caregiver's physical, psychological, and economic status. It is possible that caregivers with excessive stress often fail to provide the necessary daily care to their elder parents. In addition, there is a high likelihood that the stressed caregiver could be responsible for mistreatment.

There are several limitations that must be taken into consideration in our study. First, although the study region was a typical rural area of Hubei province and had levels of economic development and modernization comparable to those in other provinces of rural China, caution should be exercised in generalizing our findings to China's 100 million rural older residents. Furthermore, our results should not be extrapolated to populations in urban areas of China with a different social culture and lifestyle. Second, this was a cross-sectional study, and its results represented associations between variables only. It is not appropriate to make inferences regarding cause and effect among these variables. Future prospective studies are needed to explore the causal and temporal associations between the variables identified in this study. Third, information regarding elder mistreatment was obtained by participant self-report. The validity of elderly adults' answers might be distorted by recall bias. Fourth, characteristics of perpetrators such as mental illness, alcohol and other substance abuse were reported to increase the risk of elder mistreatment [32], [33], but our study considered only characteristics of the victims of mistreatment and not the abusers. Finally, the estimated prevalence rate for self-reported elder mistreatment in study sample might underestimate the actual situation as cognitively impaired adults (at greater risk of elder mistreatment) were excluded. Traditional Chinese culture has a deep-rooted idea that every family has difficulties and that outsiders should not meddle with another's family affairs. These types of issues are “family matters” that should be handled within the family. Furthermore, because the abusers are often members of the family, there is a tendency for family members to cover up the situation. In addition, there is a tendency for the victim to protect the abuser from “getting in trouble.” Consequently, it is possible that our study underestimated the occurrence of elder mistreatment in this rural Chinese community. Nevertheless, this study does provide some preliminary results about elder mistreatment and its risk factors in a Chinese rural population. In spite of these limitations, it is noteworthy that various forms of elder abuse as identified by Western countries are also present in Chinese rural areas. Some of the risk factors for elder mistreatment in this Chinese rural community are consistent with those found in Western countries.

Elder mistreatment is relatively unexplored in Chinese society. Results obtained in this study suggest that the prevalence of elder mistreatment is high in rural communities of China. This study has potential implications not only for health care professionals, but also for community policies concerning assessment, treatment, and prevention strategies. Health care professionals should pay special attention to elder adults with physical disabilities, those who are widowed, divorced, single or separated, and those with depression because they may be at an increased risk of mistreatment. Furthermore, social services agencies should be aware of risk factors for elder mistreatment and devise detection, intervention, and prevention strategies to address such mistreatment in an effort to improve the health and wellbeing of older adults.
